# Preserving Harmonic Structure in FPVS‐Oddball: A Two‐Dimensional Cluster‐Based Permutation Approach

**DOI:** 10.1111/psyp.70361

**Published:** 2026-07-17

**Authors:** Oliver Hermann, Wendy Wong Hiu Ching, George Stothart

**Affiliations:** ^1^ Department of Psychology University of Bath Bath Somerset UK; ^2^ Cumulus Neuroscience Ltd. Belfast UK

## Abstract

The fast periodic visual stimulation oddball paradigm (FPVS‐oddball), which combines multi‐input frequency tagging with an oddball design, is a powerful electroencephalography (EEG) technique for probing cognitive function. Traditionally, FPVS‐oddball analysis collapses steady‐state responses across harmonics into composite measures, which improves response detection but discards valuable information about the harmonic composition of responses. Here, we apply a two‐dimensional (sensor × harmonic) permutation test, incorporating a novel free harmonic clustering procedure that allows clustering across sensors and harmonics, thereby preserving harmonic‐related information. Using datasets on object recognition (real vs. pseudo‐objects) and line orientation discrimination, we show that the two‐dimensional approach detects condition‐specific differences in the spatial and harmonic distribution of responses that are missed by a one‐dimensional (sensor‐only) test of composite responses. For object recognition, real objects elicited stronger left‐ and right‐lateralised oddball responses at higher harmonics, consistent with additional semantic processing. For orientation discrimination, large versus small deviations elicited distinct harmonic‐specific activation patterns, reflecting qualitatively different processing. These results demonstrate that two‐dimensional cluster‐based permutation testing provides sensitivity to the spatial and harmonic distribution of FPVS‐oddball responses.

## Impact Statement

This paper introduces a two‐dimensional cluster‐based permutation approach with free harmonic clustering for FPVS‐oddball data, preserving information about the harmonic structure of responses. By considering both sensor and harmonic information, the method increases sensitivity compared with composite measures that collapse across harmonics, as has been used in previous FPVS‐oddball studies. The novel two‐dimensional approach enhances the utility of FPVS‐oddball for probing cognitive function and detecting impairments in clinical populations, such as dementia.

## 1 Introduction

### 1.1 Multi‐Input Frequency Tagging

**FIGURE 1:**
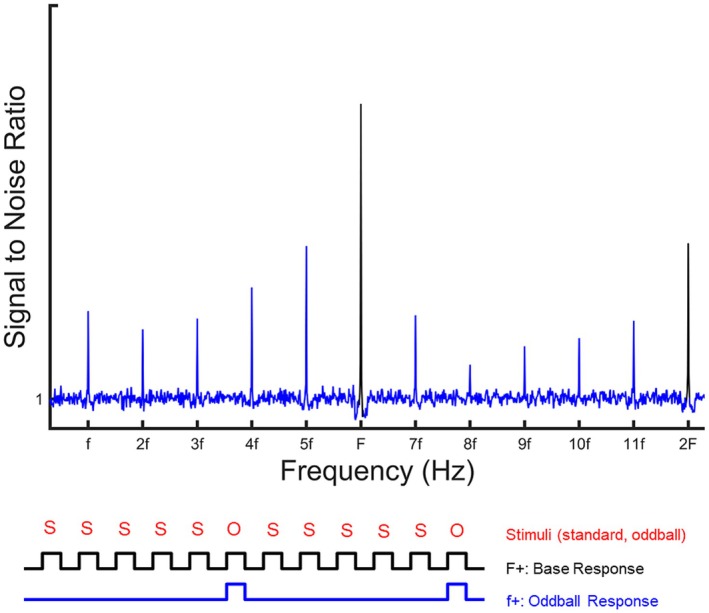
Schematic of the FPVS‐oddball design. The above panel shows an example spectral plot, illustrating the narrowband power that comprises steady state responses. F is the response at the base stimulation frequency, while the peak at 2F is its second harmonic; power at f and its harmonics is the oddball response. The below panel is a template stimuli stream where S represents standard stimuli, and O represents oddballs. The black line shows the visual steady‐state response, and the blue line shows the additional hypothesized oddball response.

Multi‐input frequency tagging is a contemporary electroencephalography technique in which different frequencies are embedded in a single stimulus presentation sequence (Braddick et al. 1986). The fast periodic visual stimulation oddball paradigm (FPVS‐oddball) combines multi‐input frequency tagging and a classic oddball design, wherein stimuli belonging to one class (standards) are repeatedly presented, with stimuli belonging to another class (oddballs) occasionally inserted into the presentation stream (Heinrich et al. 2009). This design contains two presentation frequencies: a base frequency at which the stimulus stream is presented and a lower, oddball‐specific frequency that is a submultiple of the base frequency (Figure 1). The periodically presented stimuli elicit a steady‐state neural response that is time‐locked to each stimulus (Regan 1966, 1989). Additionally, if the observer's brain can differentiate the two classes of stimuli, a neural response is specifically evoked by oddball stimuli, hereafter referred to as the oddball response. Since neural activity is elicited at specific frequencies that are defined a priori, it is natural to analyze FPVS‐oddball responses in the frequency domain, where they manifest as power at the base presentation frequency (F) and its higher harmonics, that is, 2F, 3F, etc., and the oddball presentation frequency (*f*) and its higher harmonics, that is, 2*f*, 3*f*, etc. (Norcia et al. 2015). Through the selection of stimuli, researchers can manipulate the cognitive processes required to discriminate between standards and oddballs, which has allowed the adaptation of FPVS‐oddball to measure functions, including recognition memory (Stothart et al. 2020), line orientation discrimination (Hermann et al. 2025), face processing (Rossion et al. 2015), semantic categorization (David et al. 2025; Milton et al. 2020; Stothart et al. 2017; Volfart et al. 2021), statistical learning (De Rosa et al. 2022), and lexical discrimination (Lochy et al. 2015).

### 1.2 Higher Harmonics

The distribution of signal across harmonics represents the frequency components of neural responses in the time domain: a pure sinusoid is represented by power at a single frequency, whereas a more complex, non‐sinusoidal response is represented by power across multiple higher frequencies. Historically, power at higher harmonics has been attributed to non‐linearity in stimulus presentation and/or neural responses (Norcia et al. 2015), although, evidence suggests a limited contribution of these factors. Regarding the former, comparable harmonic distributions are elicited by both imperfect sine wave (fade in/fade out) and square wave (on/off) presentation of complex stimuli (Burns et al. 1992; Dzhelyova et al. 2016; Fawcett et al. 2004; Retter and Rossion 2016). Similarly, while neural non‐linearities may contribute to the presence of higher harmonics, power at these frequencies is often comparable to noise levels, implying a limited influence (Retter et al. 2021). Therefore, the overall harmonic composition is best interpreted as being informative about the shape of the associated time series response, wherein a more complex (less sinusoidal) waveform decomposes across additional higher harmonics. The putative cause of this change in complexity is dependent on the specific task paradigm employed, although a more complex waveform is indicative of a more distributed neural response involving additional processing stages (e.g., downstream encoding of visual information) (Retter and Rossion 2016).

### 1.3 Composite Harmonic Measures in FPVS Oddball Research

In FPVS‐oddball studies, responses are generally classified by combining activity across harmonics, up to a pre‐defined threshold, wherein a harmonic is only included if the narrowband peak at its frequency is above a certain value (e.g., Rossion et al. 2015). Notably, classical steady‐state approaches typically use higher stimulation frequencies (> 8 Hz) than FPVS‐oddball, which elicit approximately sinusoidal brain responses that predominantly decompose onto the stimulated frequency. However, the lower stimulation frequencies used in FPVS‐oddball studies evoke non‐sinusoidal responses that are best represented across multiple higher harmonics (Retter and Schiltz 2025). Typically, FPVS‐oddball responses have been combined by averaging signal‐to‐noise ratio (SNR) (e.g., Barnes et al. 2021; Hermann et al. 2025; Jacques et al. 2020; Liu‐Shuang et al. 2014; Stothart et al. 2021), or summing baseline‐corrected amplitude (e.g., De Keyser et al. 2018; De Rosa et al. 2022; Rossion et al. 2023). While calculating composite values across harmonics aids the measurement, classification, and detection of responses in FPVS‐oddball and other frequency tagging paradigms (Retter et al. 2021), it discards valuable information about the harmonic composition of the evoked response, and, therefore, the complexity of the underlying neural response.

Despite the predominant use of composite scores, harmonic‐specific effects have been reported in FPVS‐oddball research. Notably, responses elicited by changes in face identity are strongest over the 3rd and 4th harmonics (Dzhelyova et al. 2016; Dzhelyova and Rossion 2014; Liu‐Shuang et al. 2014) and while harmonic profiles vary between individuals, they have been demonstrated to be stable within the same person across testing sessions (Dzhelyova et al. 2019). Indicative of an increasingly complex underlying neural response developing with age, power at additional higher harmonics has been observed in older participants using tasks designed to measure face recognition (Lochy et al. 2019), face trustworthiness discrimination (Siddique et al. 2023), and animal versus object classification (Peykarjou et al. 2024). Additionally, the scalp distribution of oddball responses elicited by a change to a recognized face differs between harmonics, with additional occipitoparietal activity present at the first harmonic, possibly reflecting a distinction between perceptual and post‐perceptual processing (Campbell et al. 2020). These findings illustrate the importance of considering the harmonic composition of an elicited response, which is lost when power at the stimulated frequency and its higher harmonics is combined by averaging or summing.

### 1.4 Cluster‐Based Permutation Tests

Cluster‐based permutation tests are widely used in EEG and MEG research to address the multiple comparison problem inherent to contrasting high‐dimensional data, which typically comprises thousands of samples, across sensors, timepoints and/or frequency bins (Maris and Oostenveld 2007). These tests are based on the assumption that true effects are likely to extend over neighboring samples, along a given dimension (Bullmore et al. 1999). By using the data's cluster structure, that is, the combined test values (e.g., *F*, *t*, etc.), of adjacent samples, as a test statistic, they control the family‐wise error rate (FWER), that is, the probability of one or more false positives when conducting multiple tests (Pernet et al. 2015).

Although cluster‐based permutation tests are designed for multi‐dimensional data, they have only been used in FPVS‐oddball research to analyze composite responses combined across harmonics, along a single dimension (sensor location) (e.g., De Rosa et al. 2022; Hermann et al. 2025; Pescuma et al. 2022; Verosky et al. 2024). In this study, we extend the approach to two‐dimensional FPVS‐oddball data (sensor × harmonic) to analyze the spatial and harmonic distribution of frequency domain responses, and crucially, retain valuable information carried by harmonic profiles.

A barrier to applying two‐dimensional cluster‐based permutation testing to FPVS data is that adjacent harmonics of a stimulated frequency are not meaningful sequential neighbors, in the manner that two adjacent sensors, timepoints, or frequency bins are. Indeed, as the presence of signal at higher harmonics simply reflects the complexity of the response in the time domain, signal manifesting at adjacent harmonics is not necessarily more likely than it manifesting at two distant harmonics. Therefore, as an assumption of clustering is that an effect is more likely to extend over adjacent samples, it is necessary to adapt the clustering procedure for multi‐harmonic FPVS‐oddball data to allow clustering of non‐adjacent harmonics. Critically, as complex, non‐sinusoidal brain responses, such as those elicited in the FPVS‐oddball paradigm, decompose across multiple harmonics, it can be inferred that signal at one harmonic of a stimulated frequency (e.g., the oddball presentation frequency), increases the likelihood of signal at other harmonics of the same frequency. Therefore, we introduce an approach, hereafter referred to as free harmonic clustering, in which clustering is permitted between any harmonic of the stimulated frequency, providing the sensor location is the same.

### 1.5 Methodological Outline

Here, we outline the procedure for implementing free harmonic clustering, before demonstrating its integration into a cluster‐based permutation test, as proposed by Maris and Oostenveld (2007). The method is demonstrated for two independent datasets: first, a previously unpublished dataset comparing object recognition to pseudo‐object perception (Supporting Information A), and second, a measure of line orientation discrimination (Hermann et al. 2025). The specific null hypothesis evaluated by, and a step‐by‐step guide for conducting, a cluster‐based permutation test is included in Supporting Information B. Additionally, we include justification for key analysis parameters.

## 2 Free Harmonic Clustering

Under free harmonic clustering, three types of clusters can form. First, spatial clusters form when selected samples correspond to spatially adjacent sensors and share the same harmonic, for example, 2F1, 2Fz, and 2F2 (electrodes F1, Fz, and F2 for the first harmonic of the stimulation frequency). Second, harmonic clusters form when selected samples come from the same sensor but different harmonics, for example, 2Fz, 4Fz, and 7Fz (electrode Fz for the first, third, and sixth harmonic of the stimulation frequency). Third, compound clusters can form between selected samples through a combination of spatial and harmonic clustering. Specifically, a cluster could form between 2F1, 2Fz, 7Fz, and 7F2.

**FIGURE 2:**
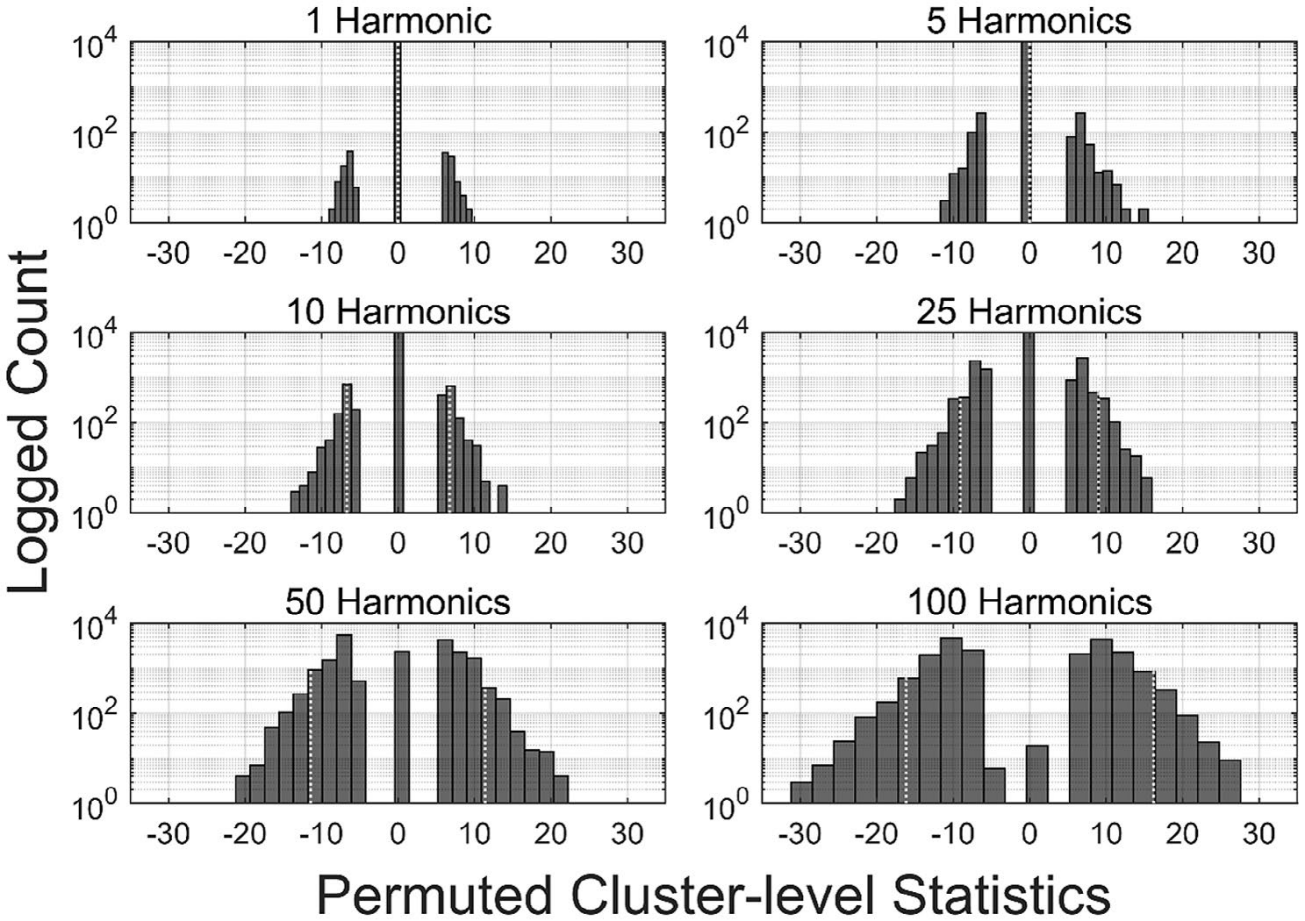
Histograms of maximum permuted cluster‐level statistics for different numbers of harmonics. White dotted line indicates the 2.5th and 97.5th percentiles, that is, the two‐tailed thresholds for critical *α* = 0.05.

Before conducting cluster‐based permutation testing, researchers must decide which harmonics to include for analysis. As the benefit of the two‐dimensional approach is its sensitivity to the harmonic composition of the measured signal, the simplest approach would be to include all harmonics of the relevant frequency, up to the frequency of any low‐pass filter applied to the data. However, due to the nature of free harmonic clustering, doing so drastically impacts the distribution of permuted maximum cluster statistics. Specifically, if the selected number of harmonics is denoted by x, then under free harmonic clustering, each sample can cluster with x−1 samples by harmonic clustering, in addition to adjacent samples through spatial clustering. As illustrated in Figure 2, under the null hypothesis, this results in the distribution of maximum cluster‐level statistics, shifting toward larger values, as more harmonics are included. Therefore, as it is well established that FPVS‐oddball effects occur across specific harmonics, a predefined threshold should be used to select harmonics. This must be independent from the permutation test, so consistent with previous FPVS‐oddball studies, for example, Liu‐Shuang et al. (2014), we recommend using z‐scores calculated from grand average data (averaged across subjects and sensors), with a cut‐off for included harmonics applied.

## 3 Analysis Pipeline and Example Datasets

### 3.1 Signal Extraction and Normalization

The following section outlines the analysis pipeline, applied here to example datasets that capture well‐established stages of visual processing: orientation processing, global shape perception, and object recognition. These are useful for both cognitive neuroscientists trying to identify the neural and functional underpinnings of visuoperception and for clinical neuroscientists trying to detect impairments.

### 3.2 Example Dataset 1: Object Recognition Versus Pseudo‐Object Perception

The previously unpublished object recognition and pseudo‐object perception conditions are outlined in full in Supporting Information A. Briefly, during object recognition, standard stimuli were images of objects from the Bank of Standardized Stimuli (BOSS) (Brodeur et al. 2014) that had been diffeomorphically scrambled beyond recognition, while oddballs were unscrambled, recognizable objects. In pseudo‐object perception, standard stimuli were diffeomorphically scrambled AI‐generated pseudo‐objects, from the IMAGINE database (Cooper et al. 2023). These images retain the low‐level perceptual features of objects, that is, texture, color, edges, and crucially, a coherent global shape, but are not recognizable. Oddball stimuli were unscrambled pseudo‐objects.

**FIGURE 3:**
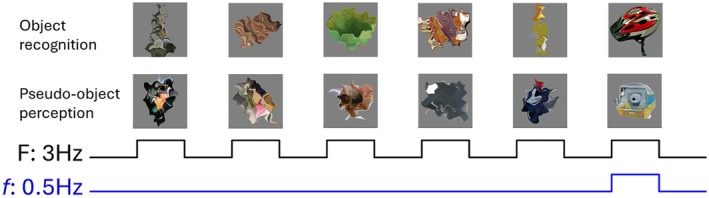
Example six‐item sequence for Object recognition (top panel) and Pseudo‐object perception (bottom panel). Black lines illustrate the hypothesized base responses, while the blue lines show oddball responses.

Nineteen participants completed both FPVS‐oddball conditions (object recognition and pseudo‐object perception) in a counterbalanced order (Figure 3). In each condition, participants viewed a series of images presented rapidly and sequentially in the center of a black surrounding square, while sat 70 cm from a screen. In both conditions, images were presented for 166.66 ms, followed by a 166.66 ms ISI before the next image appeared. Images were presented in six‐item sequences: the first five were standard stimuli (scrambled images) and sixth an oddball (an original, unscrambled image). For both conditions, the base presentation frequency was 3 Hz, and the oddball presentation frequency was 0.5 Hz. Overall, 98 six‐image sequences were presented over 196 s. Participants were instructed to keep their gaze within a surrounding square and to respond when it changed from black to red. The color change lasted 2 s and occurred 10 times per condition. This orthogonal task was included based on previous feedback that it was very difficult to continually look at the screen during completely passive stimulation. Full experimental methodology is described in Supporting Information A.

### 3.3 Example Dataset 2: Line Orientation Discrimination

**FIGURE 4:**
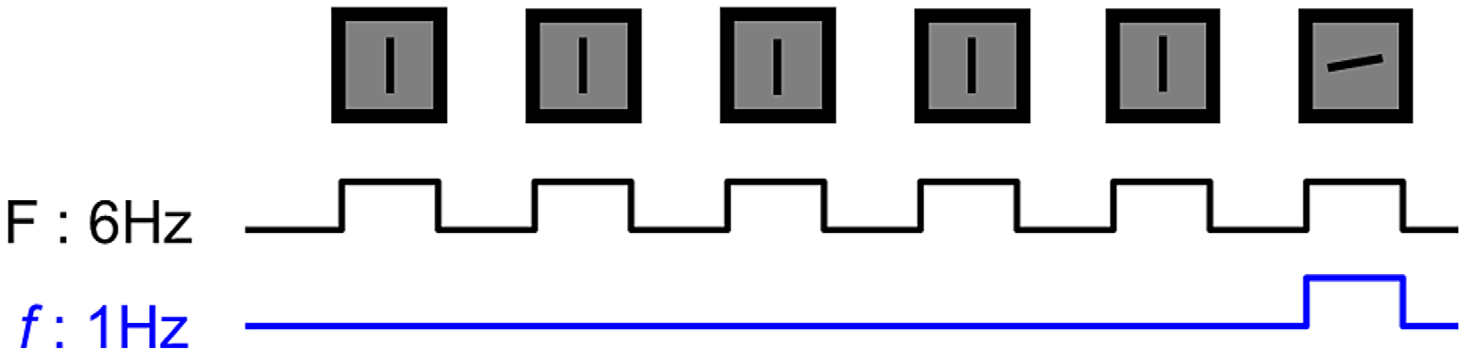
Example six‐item sequence for 80°‐line orientation discrimination task. Black lines illustrate the hypothesized base responses, while the blue lines show oddball responses.

Next, we applied the analysis pipeline to Hermann et al. (2025)'s line orientation discrimination task, in which standard stimuli were repeating vertical lines, while oddballs were lines rotated from vertical. Thirty participants completed five test conditions, in which lines were rotated by either ±1°, ±5°, ±10°, ±30°, or ±80° (Figure 4). In each condition, participants viewed a series of images, presented rapidly and sequentially in the center of a black surrounding square, while sat 70 cm from a screen. Each line was presented for 83.33 ms, followed by an 83.33 ms ISI, before the next line appeared. Lines were presented in six‐item sequences: the first five were standard stimuli (vertical lines) and the sixth an oddball (an oblique line). For both conditions, the base presentation frequency was 6 Hz, and the oddball presentation frequency was 1 Hz. Overall, 100 six‐line sequences were presented over 100 s. Participants were instructed to keep their gaze within a surrounding square and to respond when it changed from black to red. The color change lasted 2 s and occurred 10 times per condition. Full experimental methodology is detailed in Hermann et al. (2025). The previous study demonstrated qualitatively different topography of oddball responses in 80°, compared to other conditions, in which an oddball response was elicited (5°, 10°, and 30°). Therefore, here, we compare 80° to an average across 5°,10°, and 30°.

### 3.4 EEG Recording and Pre‐Processing

The following section outlines EEG recording and analysis procedures for both example datasets. EEG waveforms were sampled at 1000 Hz using a Brain Products EEG recording system (BrainVision Recorder, Vers 1.23.0001, Brain Products GmbH, actiChamp Plus), with a common FCz reference. Impedances were below 10kΩ. EEG analysis was performed offline using Brain Electrical Source Analysis software v7.0 (BESA GmbH) and MATLAB (Mathworks Inc.) BESA's automatic artifact correction (Berg and Scherg 1994) was applied to correct for blinks and eye movements. Offline, EEG data were re‐referenced to a common average reference and downsampled to 120 Hz. A 40 Hz 24 db, zero‐phase low‐pass filter was applied to prevent artifact aliasing. Epochs between 0 s and the task length (100 s for line orientation conditions; 196 s for pseudo and real object conditions) around condition onset were defined. Due to the long duration of epochs, gross artifacts, for example, those caused by large physical movements, were occasionally visible. Any artifact of ±250 μV, or greater was removed from the data and replaced with zeros. Data on either side of any removed sections were tapered to zero by applying half a Hanning window over 670 points of data. Supporting Information A details the amount of data removed by this procedure per condition. Epochs were polynomially de‐trended to remove DC and slow wave artifacts. Per subject and sensor, full condition time‐series were decomposed into the frequency domain using fast Fourier transforms. Discrete frequency bins were created for the oddball presentation frequency and its harmonics. For the pseudo‐ and real object conditions, the frequency resolution was 0.0057 Hz, while for the line orientation conditions, the frequency resolution was 0.01 Hz. These values also corresponded to the size of each frequency bin.

### 3.5 EEG Analysis

**FIGURE 5:**
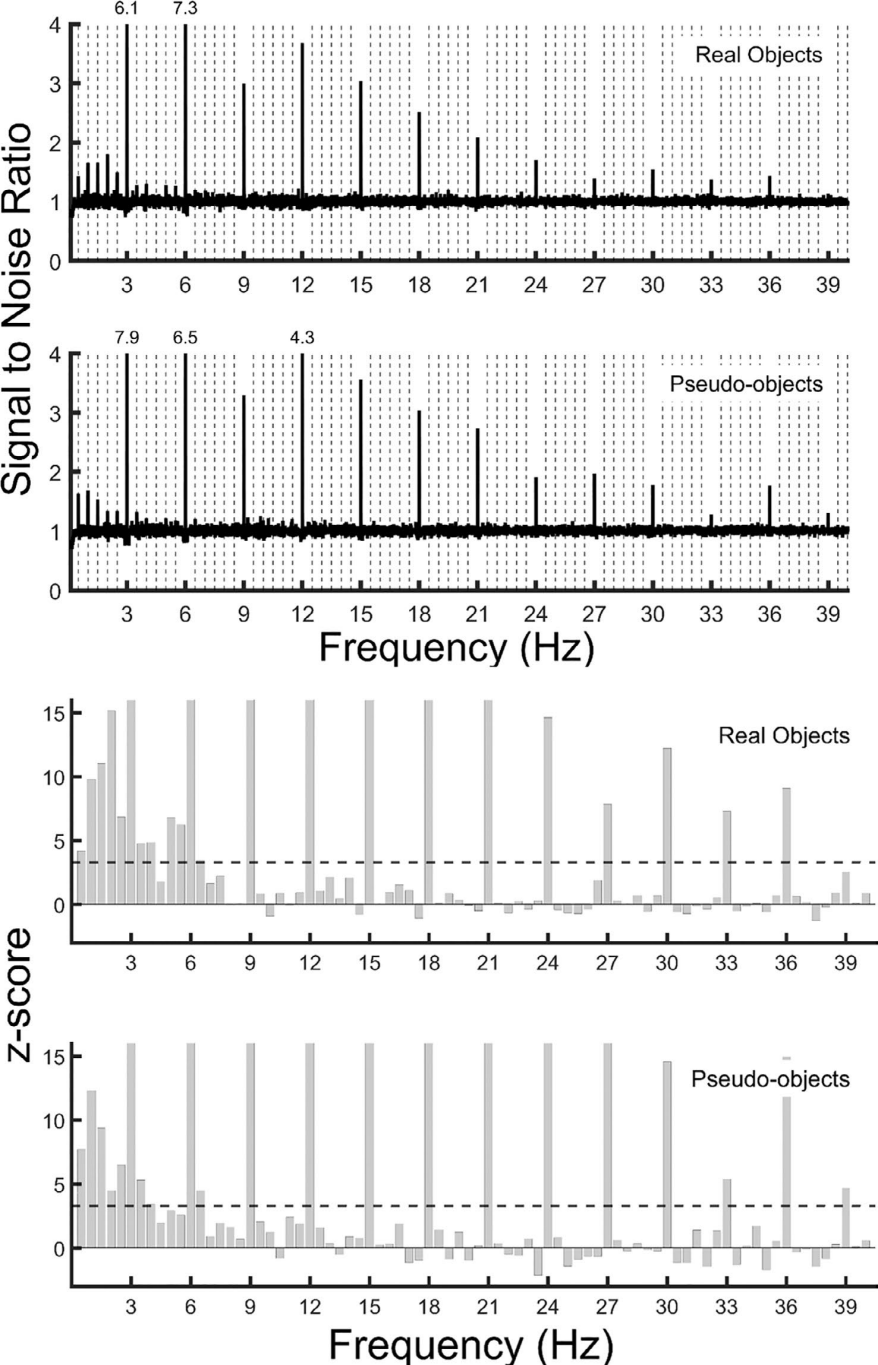
Above—Spectral plots of scalp‐average SNR, averaged across participants, per condition. Dotted lines correspond to harmonics of the oddball presentation frequency (0.5 Hz). Below—*Z*‐scored grand average amplitude at harmonics of the oddball presentation frequency (0.5 Hz). Horizontal line corresponds to the *z* = 3.29 threshold for selecting significant harmonics (Section 3.5).

Per condition, significant harmonics of the oddball presentation frequency were determined using *z*‐scores calculated from grand average data (across participants and sensors). Specifically, the *z*‐score for a given frequency bin was calculated, relative to the mean and standard deviation of the amplitude in surrounding frequency bins, in a ±0.1 Hz range, excluding the immediately adjacent bins. Included harmonics were those up to and including the highest harmonic with a *z*‐score > 3.29 (*p* = 0.001), in at least one condition. This calculation excluded the base presentation frequency and its harmonics. Significant harmonics included for each example dataset are subsequently illustrated in Figure 5 and Figure 8.

To calculate SNR, the amplitude within each frequency bin was divided by the mean amplitude across frequency bins within a ±0.1 Hz range, excluding the immediately adjacent bin on either side. These bins were excluded to mitigate potential signal spread due to the very high frequency resolution attained. To explore topographic differences while controlling for overall differences in magnitude between conditions, data were scaled by their L2 norm (McCarthy and Wood 1985), calculated as the square root of the sum of squares of all samples within each subject‐specific data structure. Each sample was then divided by this value. This normalization procedure was performed per subject and condition.

### 3.6 Cluster‐Based Permutation Testing

The cluster‐based permutation test performed here is described in Supporting Information B and follows the procedure outlined in Maris and Oostenveld (2007). We first conducted a one‐dimensional cluster‐based permutation test to investigate differences in composite oddball responses (mean SNR across included harmonics) between conditions. For this analysis, each subject‐specific data structure had a single dimension corresponding to sensor location, along which samples could cluster. Next, we performed two‐dimensional cluster‐based permutation analysis, without first averaging across harmonics. In this analysis, each subject‐specific data structure had two dimensions (sensor and harmonic), along which clusters could form, according to the clustering structure outlined in Section 2. All analyses were two‐tailed; hence, statistical significance was evaluated at Bonferroni corrected *α* = 0.05/2 = 0.025.

For both one‐dimensional and two‐dimensional cluster‐based permutation testing, the same key parameters are applied, according to the justification provided in Supporting Information B. Specifically, a paired‐samples *t*‐test was used to compare samples, the threshold for initial cluster entry was *α* = 0.01, final clusters were evaluated at *α* = 0.05/2, and 10,000 random partitions were performed. We report all clusters formed, regardless of their statistical significance, alongside the summed *t*‐values across comprising samples, and their Monte Carlo‐estimated *p*‐value and confidence interval. Additionally, we report effect size estimates using Cohen's *d*, based on the average signal per subject and condition across samples within each cluster, following the method outlined in Meyer et al. (2021) (Option 1).

## 4 Results

### 4.1 Example Dataset 1: Object Recognition Versus Pseudo‐Object Perception

#### 4.1.1 Spectral Responses

**FIGURE A1:**
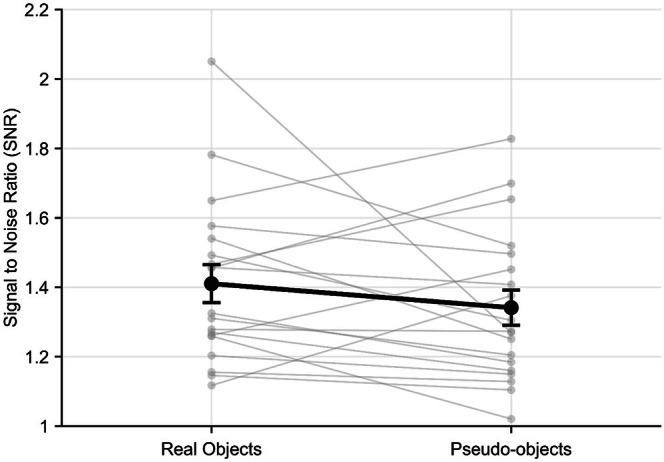
Paired observation plot for scalp‐averaged, non‐normalized data in Example Dataset 1 (Real objects vs. Pseudo‐objects). Gray lines connect individual participant data points across conditions. The bold black line represents the condition means, with error bars indicating ±1 standard error of the mean.

Clear narrowband peaks were observed at the base presentation frequency (3 Hz) and its harmonics, in both conditions. As expected, activity was elicited at the oddball presentation frequency (0.5 Hz) in both conditions (Figure 5). Using the threshold outlined in Section 3.1 (i.e., *z*‐score values that exceeded 3.29), harmonics of the oddball frequency up to and including 6.5 Hz were included for further analysis. Activity at 3 Hz and its harmonics was excluded, hence the oddball response included signal at 0.5, 1, 1.5, 2, 2.5, 3.5, 4, 4.5, 5, 5.5, and 6.5 Hz. Figure A1 contains paired observation plots of non‐normalized data for these conditions.

#### 4.1.2 One‐Dimensional (Sensor) Cluster‐Based Permutation Testing

**FIGURE 6:**
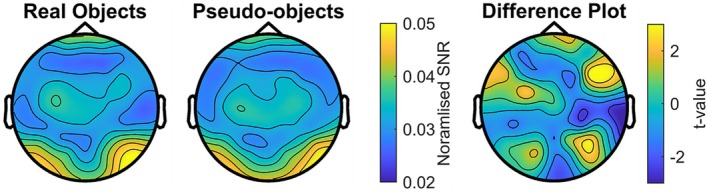
Topographic and difference plots of composite oddball responses.

One‐dimensional (sensor) cluster‐based permutation testing demonstrated no significant effect of condition (Real Objects vs. Pseudo‐objects), on composite oddball responses (mean SNR across included harmonics), scaled by their L2‐norm (Figure 6). While not providing sufficient evidence for an effect of condition (i.e., *p* < 0.025, two tailed), the difference was strongest across a two‐electrode cluster (electrodes F6 and F8), in which greater activity was elicited by real objects, *t* = 6.26, *p* = 0.031, *ci* = (0.029, 0.033), Cohen's *d* = 0.82.

#### 4.1.3 Two‐Dimensional (Sensor × Harmonic) Cluster‐Based Permutation Testing

**FIGURE 7:**
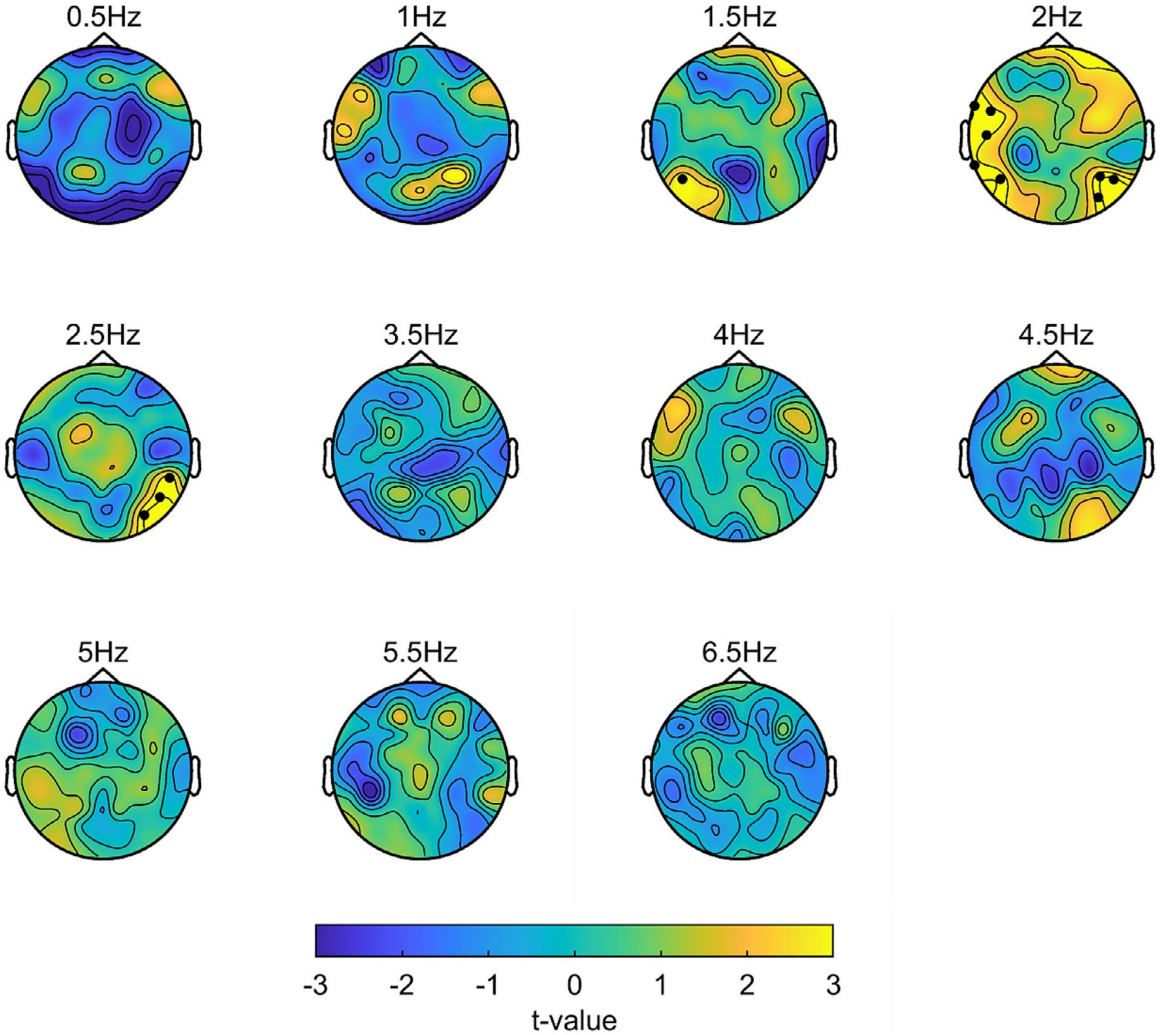
Difference plots (Real objects versus pseudo‐objects) of oddball responses per harmonic. Dots illustrate electrodes within clusters that reached statistical significance (*α* = 0.05/2). Black dots illustrate positive‐going clusters (Real Object > Pseudo‐object), while white dots show negative‐going clusters (Pseudo‐object > Real Object).

Two‐dimensional (sensor × harmonic) cluster‐based permutation testing demonstrated a significant difference in L2‐normalized oddball responses between conditions (Real Objects vs. Pseudo‐objects) (Figure 7). This difference was most pronounced across two posterior positive‐going compound clusters, where stronger activity was elicited by real objects. First, a right‐lateralised cluster formed across the 3rd and 4th harmonics (2 Hz and 2.5 Hz), spanning parietal, occipito‐parietal, and temporo‐parietal electrodes, *t* = 21.06, *p* = 0.0128, *ci* = (0.0117, 0.0139), Cohen's *d* = 1.35. Second, a left‐lateralised cluster formed across the 2nd and 3rd harmonics (1.5 and 2 Hz), spanning parietal, temporo‐parietal, temporal, and frontotemporal electrodes, *t* = 19.26, *p* = 0.0180, *ci* = (0.0167, 0.0193), Cohen's *d* = 1.61.

Additionally, two negative‐going spatial clusters formed, which did not provide evidence for a difference between conditions. First, a predominantly right‐lateralised cluster formed at the oddball presentation frequency (0.5 Hz), spanning occipital, occipito‐parietal, and parietal electrodes, *t* = 17.28 *p* = 0.0273 *ci* = (0.0257, 0.0289), Cohen's *d* = 0.98. Second, a left‐lateralised cluster formed at the oddball presentation frequency (0.5 Hz), spanning central and frontocentral electrodes, *t* = 10.10 *p* = 0.1150 *ci* = (0.1119, 0.1181), Cohen's *d* = 0.99.

#### 4.1.4 Summary

In summary, the two‐dimensional (sensor × harmonic) cluster‐based permutation test revealed qualitatively distinct distributions of neural responses to real versus pseudo‐object oddballs—differences that the one‐dimensional (sensor‐only) test failed to detect. More pronounced activity was elicited by real objects across two lateralised clusters, spanning the 2nd to 4th harmonics. Notably, difference plots per harmonic, alongside nonsignificant clusters at the oddball presentation frequency, suggest a reversal in response patterns at posterior sensors: pseudo‐object oddball generally elicited stronger activity at the oddball presentation frequency and its 1st harmonic, whereas real objects evoked stronger responses at the 2nd to 4th harmonics. No significant difference in response distribution was detected by the one‐dimensional analysis, although a marginally nonsignificant difference was revealed which was most pronounced over a right‐sided frontal cluster (F6/F8), at which stronger activity was elicited by real objects. While this difference was present at the oddball presentation frequency, early harmonics (1st to 3rd) and at the 6th harmonic, this did not form a cluster.

### 4.2 Example Dataset 2: Line Orientation Discrimination

#### 4.2.1 Spectral Responses

**FIGURE 8:**
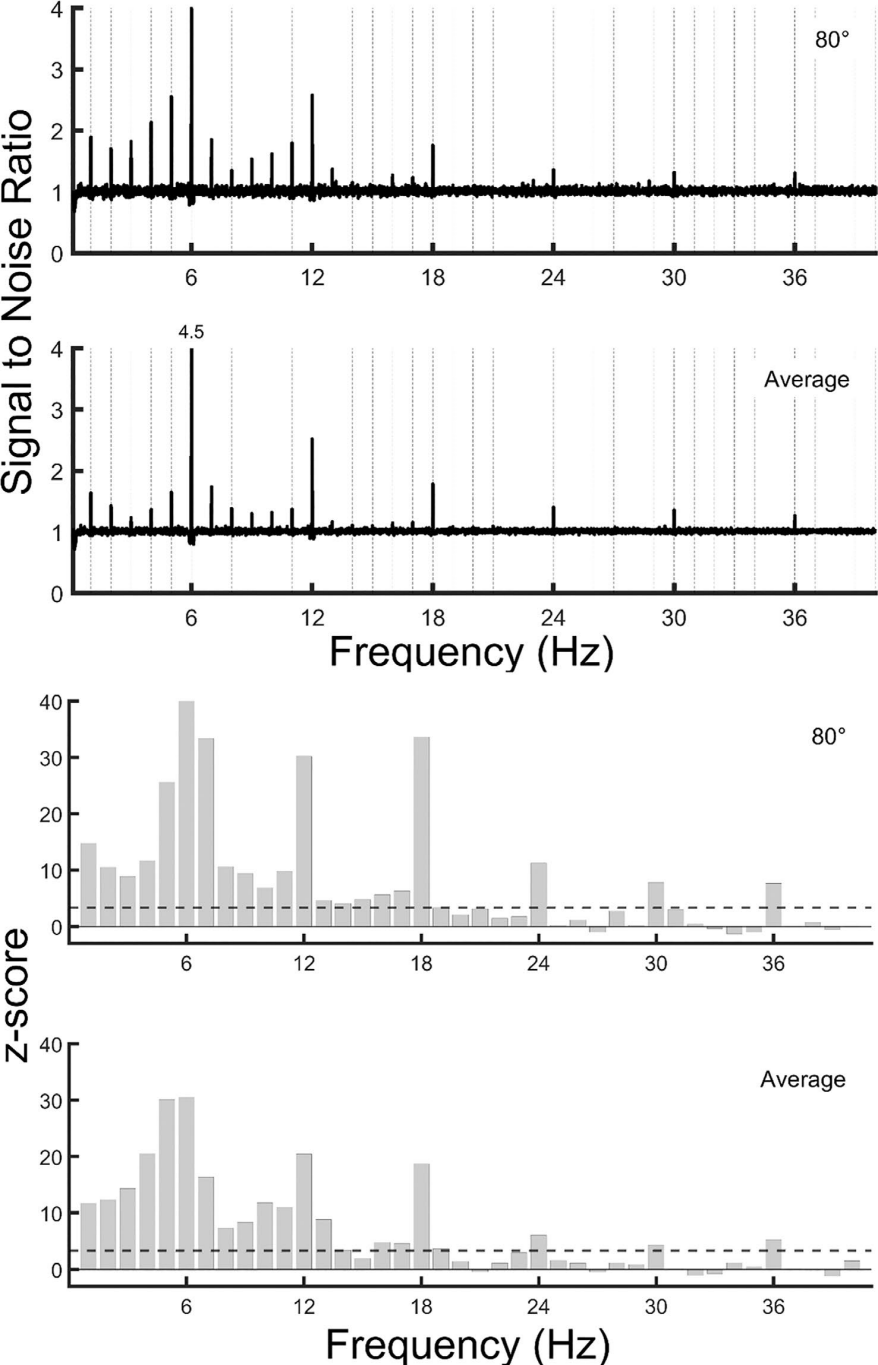
Above—Spectral plots of scalp‐average SNR, averaged across participants, per condition. Dotted lines correspond to harmonics of the oddball presentation frequency (1 Hz). Below—*Z*‐scored grand average amplitude at harmonics of the oddball presentation frequency (1 Hz). Horizontal line corresponds to the *z* = 3.29 threshold for selecting significant harmonics (Section 3.5).

**FIGURE A2:**
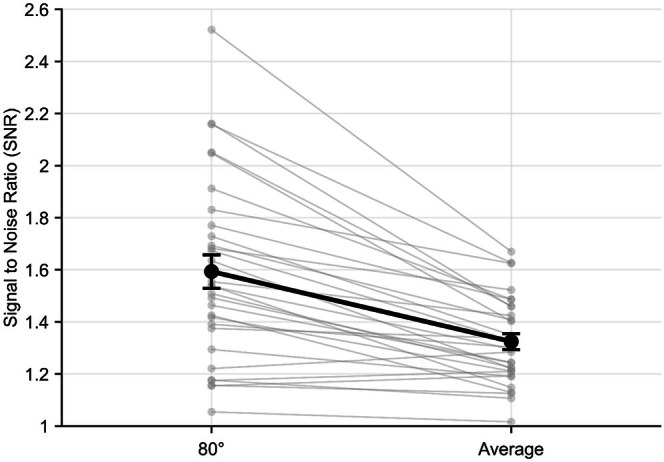
Paired observation plot for scalp‐averaged, non‐normalized data in Example Dataset 2 (80° vs. Average). Gray lines connect individual participant data points across conditions. The bold black line represents the condition means, with error bars indicating ±1 standard error of the mean.

Clear narrowband peaks were observed at the base presentation frequency (6 Hz) and its harmonics in 80°, and in data averaged across 5°, 10°, and 30°. As expected, activity was elicited at the oddball presentation frequency (1 Hz) in both conditions (Figure 8). Using the threshold outlined in Section 3.1, that is, *z*‐score values that exceeded 3.29, harmonics of the oddball frequency up to and including 19 Hz were included for further analysis. Activity at 6 Hz and its harmonics was excluded; hence, the oddball response included signal at 1, 2, 3, 4, 5, 7, 8, 9, 10, 11, 13, 14, 15, 16, 17, and 19 Hz. Figure A2 contains paired observation plots of non‐normalized data for these conditions.

#### 4.2.2 One‐Dimensional (Sensor) Cluster‐Based Permutation Testing

**FIGURE 9:**
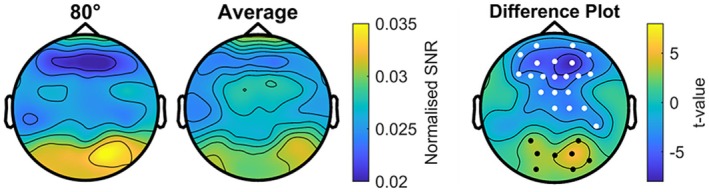
Topographic and difference plots of composite oddball responses. Dots correspond to electrodes within clusters that reached statistical significance (*α* = 0.05/2). Black dots illustrate positive‐going clusters (80° > Average), while white dots show negative‐going clusters (Average > 80°).

One‐dimensional (sensor) cluster‐based permutation testing demonstrated a significant effect of condition (80° vs. Average) on composite oddball responses (mean SNR across included harmonics), scaled by their L2‐norm (Figure 9). A positive‐going cluster formed, spanning occipital, parietal, and occipitoparietal electrodes, in which activity was stronger in 80°, *t* = 28.33, *p* < 0.001, *ci* = (< 0.001, < 0.001) Cohen's *d* = 1.20.

A large negative‐going cluster formed, spanning bilateral frontal, central, frontocentral, and right‐lateralised centroparietal electrodes, in which activity was stronger in Average, *t* = 87.01, *p* < 0.001, *ci* = (< 0.001, < 0.001), Cohen's *d* = 1.35.

#### 4.2.3 Two‐Dimensional (Sensor × Harmonic) Cluster‐Based Permutation Testing

**FIGURE 10:**
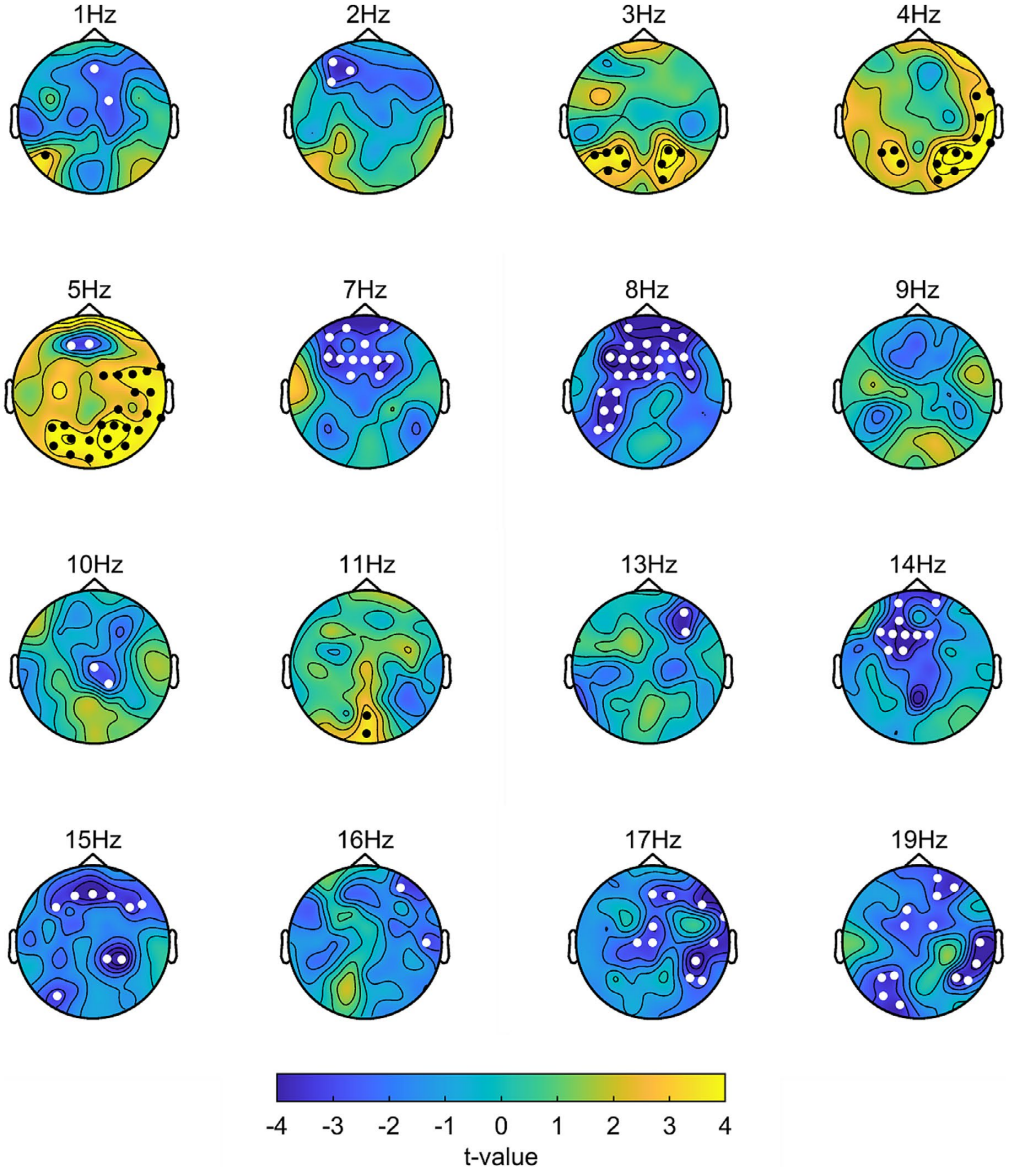
Difference plots (80° versus Average) of oddball responses per harmonic. Dots illustrate electrodes within clusters that reached statistical significance (*α* = 0.05/2). Black dots illustrate positive‐going clusters (80° > Average), while white dots show negative‐going clusters (Average > 80°).

Two‐dimensional (sensor × harmonic) cluster‐based permutation testing demonstrated a significant difference in L2‐normalized oddball responses between conditions (80° vs. Average) (Figure 10). This difference was most pronounced across one positive‐going and one negative‐going compound cluster. First, a bilateral cluster formed, where activity was more pronounced in 80°, across the oddball presentation frequency, and its 2nd, 3rd, 4th, and 9th harmonic (1, 3, 4, 5, and 10 Hz), spanning predominantly occipital, parietal, and occipitoparietal electrodes, as well as a small number of right‐sided temporal, frontocentral, and frontotemporal electrodes at the 3rd and 4th harmonic (4 and 5 Hz), *t* = 189.66, *p* < 0.001, *ci* = (< 0.001, < 0.001), Cohen's *d* = 1.87. Second, a large cluster formed wherein activity was more pronounced in Average, across almost all harmonics. This spanned most of the scalp, but was most consistently across frontal, central, and temporal electrodes, almost entirely sparing occipital electrodes, *t* = 334.66, *p* < 0.001, *ci* = (< 0.001, < 0.001), Cohen's *d* = 3.12.

#### 4.2.4 Summary

One‐dimensional (sensor) cluster‐based permutation testing demonstrated a clear difference in the distribution of oddball responses elicited by deviations of 80°, compared to small deviations (average of 5°, 10°, and 30°). This was characterized by an anterior–posterior split in the response topography: 80° elicited stronger activity over occipito‐parietal regions, whereas smaller deviations produced greater fronto‐central responses. Two‐dimensional analysis (sensor × harmonic) provided additional insight into this, demonstrating that greater occipito‐parietal activation for 80° was most pronounced at earlier harmonics. In contrast, increased frontocentral activation for smaller orientation changes spanned a broader range of mostly later harmonics, including the 6th (7Hz) and 7th (8Hz) harmonics. Notably, for some later harmonics (15, 17 and 19Hz), occipito‐parietal activation was greater for smaller deviations. In summary, both one‐ and two‐dimensional analyses suggest a different distribution in oddball responses between conditions, for larger versus smaller orientation changes, with the two‐dimensional test providing additional sensitivity to the harmonic composition of these differences.

## 5 General Discussion

### 5.1 Summary

This study demonstrated the utility of a two‐dimensional cluster‐based permutation test for analyzing FPVS‐oddball data, incorporating free‐harmonic clustering. Here, we applied the method to two example FPVS‐oddball datasets: a comparison of real object recognition versus pseudo‐object perception and a line orientation discrimination task. In the object task, the two‐dimensional test detected significant differences between responses elicited by real and pseudo‐objects that were not identified using a one‐dimensional approach. In the orientation discrimination task, both tests revealed significant differences in the distribution of responses for larger (80°) vs. smaller deviations (average of 5°, 10°, and 30°); however, the two‐dimensional analysis revealed additional information relating to scalp topography and harmonic distribution. Additionally, the two‐dimensional permutation test revealed much larger effect sizes than the one‐dimensional test for both datasets, suggesting that one‐dimensional analysis may be masking the true effect magnitude due to its insensitivity. Overall, two‐dimensional cluster‐based permutation testing offers increased sensitivity to the harmonic distribution of FPVS‐oddball responses, retaining vital information about the shape and complexity of the evoked response discarded by one‐dimensional approaches.

### 5.2 What Neural Processes Do Higher Harmonic Responses Represent

Frequency‐domain signal at higher harmonics of a stimulation frequency indexes a more complex evoked waveform, associated with a more distributed underlying neural response. While the underlying mechanisms producing a condition−/ group‐specific increase in signal at higher harmonics are task‐dependent, we propose it acts as a proxy representation of additional stages of neural processing. Importantly, individual harmonics responses are interrelated and do not capture independent neural responses (Zhou et al. 2016), hence it is not possible to attribute signal at single harmonics (e.g., the 2nd or the 5th) to specific neuronal processes. The subsequent subsections consider potential interpretations of harmonics effects for the two example datasets used here.

#### 5.2.1 Example Dataset 1: Real Object Recognition Versus Pseudo‐Object Perception

Real object oddballs elicited more pronounced oddball responses than pseudo‐objects at left‐lateralised temporo‐parietal electrodes at the 2nd and 3rd harmonic and right‐lateralised occipito‐parietal activation at the 3rd and 4th harmonic, evidencing that real object oddballs evoked additional neural processing. While both pseudo and real object oddballs could be differentiated from standards based on their coherent global shape, only real objects conveyed semantic meaning. Briefly, object recognition is a two‐stage process characterized by an initial, mostly feed‐forward sweep of neural activation along the occipitotemporal ventral visual stream, during which perceptual features are encoded (Milner and Goodale 2008). Subsequently, a second stage occurs during which these perceptual representations are integrated with stored semantic representations, through recurrent connectivity between the posterior ventral temporal cortex and the anterior temporal lobe (Clarke et al. 2018; Von Seth et al. 2023). Crucially, only real objects that corresponded to stored semantic representations could undergo this additional stage of recurrent processing. Therefore, we cautiously propose that additional activation evoked by real objects at higher harmonics (2nd–4th) reflects semantically mediated processing. This interpretation is supported by the scalp distribution of effects; more pronounced responses to real objects were recorded over electrodes consistent with temporal lobe activation.

#### 5.2.2 Example Dataset 2: Line Orientation Discrimination

Two‐dimensional cluster permutation analysis demonstrated that responses to larger deviations in orientation were relatively more pronounced at occipital and parietal electrodes, at lower harmonics, while responses to smaller deviations were relatively more pronounced at frontal and central electrodes, at higher harmonics. As initially proposed in Hermann et al. (2025), the distinct activation patterns associated with 80° may reflect qualitative differences in how the brain processes change. Specifically, for smaller deviations (up to ~40°), the brain updates its existing model to best represent the tilted line, whereas larger deviations lead to the adoption of a new model that better represents the tilted line (Smout et al. 2019). The current two‐dimensional cluster‐based permutation test results suggest that model updating is represented by a more complex frequency‐domain response, decomposing across multiple higher‐order harmonics, whereas model adoption is captured by a simpler, more focal harmonic distribution, concentrated at lower‐order harmonics.

### 5.3 Application in FPVS‐Oddball Research

Previous FPVS‐oddball studies have typically combined responses across harmonics by averaging or summing, thereby losing sensitivity to important information about the complexity and shape of the evoked time‐series waveform. Here, we integrate free harmonic clustering, a novel method that combines responses across two dimensions (sensor × harmonic), within a cluster permutation test, retaining this crucial aspect of the data. For cognitive neuroscientists, this technique can be applied to FPVS‐oddball tasks designed to measure any cognitive function, providing greater sensitivity to the neural activity and processes driving oddball responses. For clinical neuroscientists measuring oddball responses in pathological populations such as Alzheimer's Disease (David et al. 2025; Stothart et al. 2021, 2025), the harmonic composition of responses is an additional dimension along which patients may differ from controls, potentially aiding detection of cognitive impairment linked to neural pathology. In summary, a two‐dimensional approach that considers response distribution across both sensors and harmonics provides clear advantages and therefore should be incorporated into cognitive as well as clinical neuroscientific research.

### 5.4 The Impact of Stimulation Frequency

In principle, the technique for two‐dimensional cluster‐based permutation testing of FPVS‐oddball data introduced here could be applied to tasks designed to measure any cognitive function. However, an important consideration must be that the response evoked by individual stimuli is not independent of the stimulation frequency, and therefore, as this frequency is changed, the shape of the evoked response also changes. Additionally, as the stimulation frequency is increased, responses evoked by successive stimuli overlap more, leading to response superposition (Heinrich 2010). As a consequence of this, the complexity of the time‐domain waveform is decreased; hence responses decompose across fewer harmonics (Retter et al. 2021). Therefore, use of this method should be limited to comparisons between conditions where the base and oddball stimulation frequencies are identical, excluding the special case where stimulation frequency is the independent variable of interest.

### 5.5 Multiple Comparison Considerations

Applying the cluster‐based permutation test, the FWER is controlled at the level of the data structure, that is, weakly, hence inferences cannot be made about individual samples (sensor × harmonic combinations) (Sassenhagen and Draschkow 2019). An alternative technique offering strong FWER control is to compare each sample‐wise test statistic to maximum statistics permuted under the null distribution (Holmes et al. 1996; Nichols and Holmes 2002). However, this method is conservative and lowers power, reducing sensitivity to true effects (Groppe et al. 2011a, 2011b). A promising approach is the cluster depth method (Frossard and Renaud 2021, 2022), which evaluates sample‐wise test statistics against a multivariate null distribution, using Troendle (1995)'s algorithm, retaining statistical power yet allowing sample‐wise inferences to be made. However, this requires a linear clustering structure, wherein sample *n* can only cluster with sample *n + 1* or sample *n*−1, which is not the case for spatial or harmonic clustering. Therefore, to maintain both statistical power and an appropriate clustering structure for multi‐sensor/multi‐harmonic data, cluster‐based permutation testing with weak FWER control, remains the most suitable option.

In the cluster‐based permutation approach used in this study, researchers must select an initial threshold for sample‐wise comparison, based on which clusters form. Although the choice of this threshold does not affect the test's validity, providing it is consistently applied to both observed and permuted data, its value strongly impacts the clusters formed. An overly liberal threshold may produce large, diffuse clusters, that span much of the data structure, reducing specificity, while an overly conservative threshold may lead to no clusters forming, reducing sensitivity. An approach that addresses this issue and avoids arbitrary threshold selection is threshold free cluster enhancement (TFCE), which enhances or suppresses sample signal, based on its intensity and its support from surrounding samples. The altered signal is then evaluated against the distribution of maximum permuted statistics, under the null hypothesis (Mensen and Khatami 2013; Smith and Nichols 2009). While removing the researcher bias of threshold selection is appealing, for it to be applied to two‐dimensional (sensor × harmonic) FPVS‐oddball data, a decision must be made regarding the relative weighting of support from neighboring sensors versus neighboring harmonics, accounting for the effect of the free harmonic clustering structure.

### 5.6 Wider Application

A key strength of the permutation test is that input data do not need to follow a specific distribution (e.g., normal); indeed, the only requirement is that data are ordinal. Therefore, while two‐dimensional cluster‐based permutation tests, and the incorporated free harmonic clustering, were used in this study to analyze FPVS‐oddball data, it could be applied to a wide range of datasets, providing a logical neighboring structure can be defined, along which adjacent effects can correlate. Naturally, this analysis method could be applied to other SSVEP paradigms, which require the quantification of frequency‐domain responses (e.g., designs in which changes to local and global stimulus features are given different frequency tags (Braddick et al. 2005; Norcia et al. 2015)). Within study designs that modulate different stimuli elements at distinct frequencies, power can also manifest at intermodulation frequencies, which are the sum of integer multiples of the stimulation frequencies (Gordon et al. 2019). The free harmonic clustering approach would enable analysis to consider non‐adjacent frequencies (in this case, the stimulation frequency and the intermodulation frequency) as neighbors, providing additional sensitivity to experimental effects.

### 5.7 Conclusion

In summary, we introduce free harmonic clustering, a novel clustering structure that enables a two‐dimensional analysis of FPVS‐oddball data. This method offers crucial sensitivity to the spatial and harmonic distribution of frequency‐domain responses that is lost using traditional approaches and should be incorporated into both cognitive and clinical neuroscience research.

## Author Contributions

**George Stothart:** supervision, resources, conceptualization, funding acquisition, writing – review and editing, project administration. **Oliver Hermann:** conceptualization, investigation, writing – original draft, methodology, validation, visualization, writing – review and editing, software, formal analysis, project administration, data curation, resources, supervision. **Wendy Wong Hiu Ching:** methodology.

## Funding

This work was supported by BRACE, UK registered charity no: 297965, grant number: BR2250.

## Consent

Informed consent was obtained from all individual participants included in the study.

## Conflicts of Interest

G.S. is current employed by The University of Bath and Cumulus Neuroscience Ltd. Cumulus Neuroscience Ltd had no involvement in this study. All other authors have no competing interests to declare.

## Supporting information


**Data S1:** Supporting Information.

**Data S2:** Supporting Information.

## Data Availability

The data and analysis code that support the findings of this study are openly available in GitHub at https://github.com/users/oliver‐hermann1/projects/1.
