# Supplementary material for: Preserving Harmonic Structure in FPVS‐Oddball: A Two‐Dimensional Cluster‐Based Permutation Approach
Source: Psychophysiology. 2026 Jul 17;63(7):e70361. doi: 10.1111/psyp.70361 (PMC13379596; doi:10.1111/psyp.70361)
Supplement: Supplementary file 1 — Data S1: Supporting Information. [file PSYP-63-e70361-s002.docx]

# Supplementary Material A

Real object recognition versus pseudo-object perception methodology

## Design

All participants completed two FPVS-oddball conditions, Object Recognition and Pseudo-object Perception. In both conditions, standard stimuli were diffeomorphically scrambled images and oddball stimuli were original, unscrambled images. In the object recognition condition, stimuli were images of recognisable everyday objects, while in the pseudo-object perception condition, they were AI-generated pseudo-objects. The dependent variable was the SNR at the oddball presentation frequency and its harmonics.

## Participants

Twenty adults from the University of Bath student pool were recruited for the study. One participant was excluded due to excessively noisy EEG recordings. The demographics of the remaining 19 participants were: mean age = 24 years (SD=5); number of females = 13; mean visual acuity (LogMAR) = -0.01 (SD=0); mean contrast sensitivity = 2 (LogCS=0.13).

All participants self-reported that they were neurologically healthy and had normal or corrected-to-normal vision. All participants gave informed written consent before the study and could withdraw at any time. All study procedures were performed in compliance with relevant laws and international guidelines, including privacy rights for human subjects. Ethical approval for the study was obtained from the University of Bath Psychology Research Ethics Committee (PREC reference no.: 22 151).

## Stimuli

## Object Recognition

Both standard and oddball stimuli used colour images taken from the Bank of Standardised Stimuli (BOSS), an image database containing everyday objects and items (Brodeur et al., 2014). Images of living items were removed to ensure that a systematic semantic or colour difference between oddball and standard stimuli was not inadvertently introduced. Additionally, in cases in which the database contained multiple exemplars of the same object, a single item was chosen by the experimenter. All images subtended 8° visual angle, with the central image cropped against a grey background. Stimuli were presented within a black surrounding square that subtended 9.5° visual angle.

## Standards

Standard stimuli were diffeomorphically scrambled beyond recognition, using a freely available MATLAB function (https://github.com/rhodricusack/diffeomorph). Crucially, diffeomorphic scrambling maintains low-level perceptual features of images, e.g., colour, texture, while disrupting global shape and removing semantic meaning (Stojanoski and Cusack, 2014). The following process was used to ensure scrambling beyond recognition; first, images were progressively scrambled, generating 60 images, with differing amounts of scrambling (script parameters, maxdistortion = 120, nsteps = 30). A volunteer, naïve to the study aim, viewed each object’s images from most to least scrambled and indicated the point at which the object became recognisable. Most objects became recognisable between 10 and 20 scrambling steps from the original. Any images that were recognisable at a greater number of steps than this were excluded. A second volunteer then viewed only the 20-step scrambled images and was asked whether they could identify the object. Any correctly identified image was also removed.

Per participant, 240 images were randomly selected from the overall set as standard stimuli. This ensured there were enough stimuli that each image could be presented twice. For the first presentation, an image was randomly chosen from the set of 240, then removed from the pool. After all 240 images were shown once, the random selection process repeated for the second presentation.

## Oddballs

Per participant, the oddball images were original versions of the scrambled images not used as standards. Out of this set of 137 images, 48 were randomly selected as oddball stimuli. As was the case for standard stimuli, each oddball image was randomly selected from a separate pool, then removed after being presented. Once all oddball images had been shown once, the random selection process repeated for the second presentation.

## Pseudo-object Perception

Standard and oddball stimuli used novel objects from the IMAGINE dataset (Cooper et al., 2023), a validated set of 400 artificially generated images of plausible yet non-existent objects. Crucially, these images retain the perceptual features of objects (i.e., texture, colour, edges) without being recognisable. A volunteer naïve to the intentions of the study, was asked to look through each image and state whether they recognised any object. Items falsely identified as real objects were excluded, leaving a final set of 295 pseudo-objects.

## Standards

Standard stimuli were diffeomorphically scrambled, using the same parameters as Object Recognition, i.e., scrambling through 20 steps (script parameters, maxdistortion = 120, nsteps = 30). As was the case in Object Perception, 240 images were randomly selected from the overall set as standard stimuli, ensuring each image could be presented twice. The randomised presentation order followed the same logic as Object Recognition.

## Oddballs

##

Per participant, the oddball images were the unscrambled version of the 55 images not used as standards.

## Procedure

## Baseline Visual Assessment

Before the FPVS tasks, subjects completed the Freiburg Visual Acuity and Contrast Task (Bach, 1996).

## FPVS-Oddball

This resulted in a 3Hz stimulus presentation frequency, with oddballs presented at 0.5Hz. Participants were instructed to keep their gaze within a surrounding square and to respond when it changed from black to red. The colour change lasted two seconds and occurred ten times per condition.


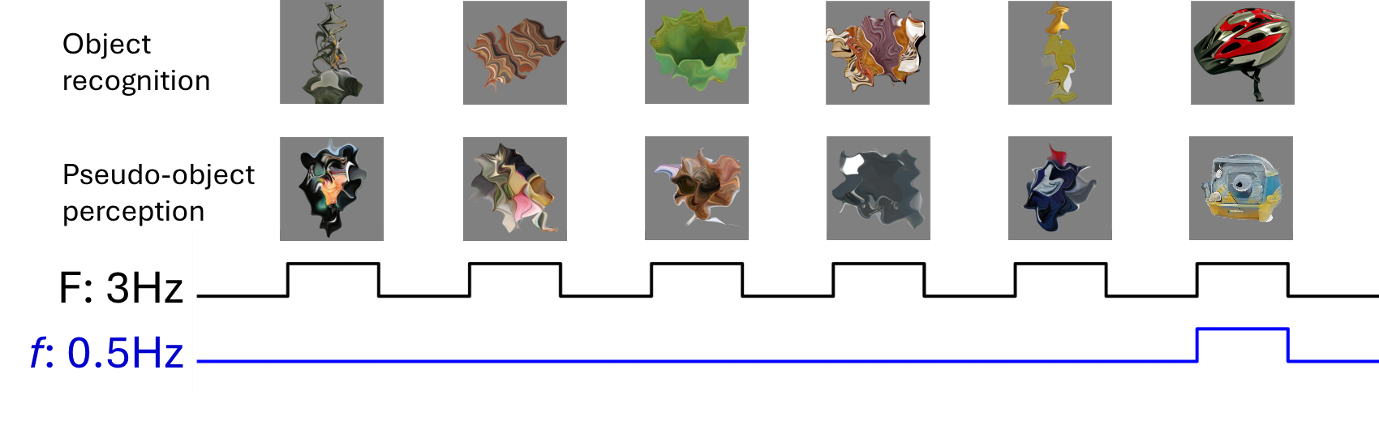


**Fig X.** Example six-item sequence for Object recognition (top panel) and Pseudo-object perception (bottom panel). Black lines illustrate the hypothesised base responses, while the blue lines show oddball responses.

## EEG Recording and Analysis

## EEG waveforms were sampled at 1000Hz using a Brain Products EEG recording system (BrainVision Recorder, Vers 1.23.0001, Brain Products GmbH, actiChamp Plus), with a common FCz reference. Impedances were below 10kΩ.

. Across participants, conditions and electrodes, the mean percentage of data removed by this procedure was 0.004 % (SD = 0.0029 %) during Object Recognition, and 0.001 % (SD = 0.003 %) during Pseudo-object perception.

Fourier transforms were used to calculate the amplitude within each frequency bin, per participant, condition, and electrode. Frequency bins were created containing the exact oddball presentation frequency (0.5Hz) and its harmonics, including the base presentation frequency (3Hz). To calculate SNR for a given frequency bin, the amplitude within that bin was divided by the mean amplitude across surrounding frequency bins in a ± 0.1Hz range, excluding immediately adjacent bins. These bins were excluded to mitigate potential signal spread due to the very high frequency resolution attained (0.0051 Hz).
