# Supplementary material for: Preserving Harmonic Structure in FPVS‐Oddball: A Two‐Dimensional Cluster‐Based Permutation Approach
Source: Psychophysiology. 2026 Jul 17;63(7):e70361. doi: 10.1111/psyp.70361 (PMC13379596; doi:10.1111/psyp.70361)
Supplement: Supplementary file 2 — Data S2: Supporting Information. [file PSYP-63-e70361-s001.docx]

**Supplementary Materials B**

Cluster-based permutation test details

## Null hypothesis formulation

Permutation tests evaluate the null hypothesis that all subject-specific data structures, $D_{r}$, have equal probability distributions, regardless of their experimental condition, hence are exchangeable. For between-subject designs, if these probability distributions are expressed as $f(D_{r}=d_{r})$, abbreviated to $f(D_{r})$, then the null hypothesis is expressed as: $f\left( D_{1} \right)=f\left( D_{2} \right)=\ldots=f(D_{n})$. In the case that $f\left( D_{1} \right)\neq\ldots\neq f\left( D_{n} \right)$, the null hypothesis of exchangeability can be rejected. For within-subject designs, the permutation test evaluates the null hypothesis that each subject’s data structures have equal probability distributions, irrelevant of condition. Let the data structures for the *r*th subject, for each condition, be denoted $D_{r1}$and $D_{r2}$. The joint probability distribution is therefore given by $f(D_{r1 ,}$ $D_{r2}$). Under the null hypothesis, this joint distribution is exchangeable, i.e., $f(D_{r1}, D_{r2}=D_{r2}, D_{r1})$, which implies that the marginal distributions are also equal, i.e., $f\left( D_{r1} \right)=f(D_{r2})$. In the case that $f\left( D_{r1} \right)\neq f(D_{r2})$, the null hypothesis of exchangeability can be rejected.

It is vital to consider the precise null hypothesis evaluated by the permutation test, as it is for this that the FWER is controlled at a critical α. As the null hypothesis relates to the entire data structure, the FWER is only controlled at the level of the data structure and not for individual samples. So, if the null hypothesis is rejected, one can conclude an effect of experimental condition on the probability distribution of some samples, but cannot make inferences regarding the loci of effects with the confidence associated with the critical α. The control of the FWER for the entire data structure, under a global null hypothesis offered by the permutation test, stands in contrast to the user’s typical interest in the loci of effects. As enacting strong control of the FWER rate at the level of individual samples dramatically reduces power, a more liberal localisation method, which is outlined in full below, is incorporated.

## Step-by-step guide

**Step 1** - For each sample, compare the recorded signal between conditions, using the chosen test statistic. While for our example datasets, paired-samples *t*-tests were applied, the procedure is agnostic to the test statistic used.

**Step 2** – Select samples for which the test statistics resulting from the between-condition comparison, exceed a predefined threshold (e.g., α ≤ 0.01).

**Step 3** – Cluster selected samples according to the chosen clustering scheme, e.g., free harmonic clustering. In the case of a two-tailed statistical test, clustering is performed separately for positive and negative test values and adjacent selected samples can only cluster if the direction of the effect is the same.

**Step 4** – Cluster-level statistics are calculated by summing test statistics within each cluster.

**Step 5 –** For one-tailed test statistics, select the largest cluster-level statistic. For two-tailed tests, select the cluster-level statistic that is largest in absolute value. The null hypothesis of equality of subject-specific data structures is evaluated using this test statistic.

**Step 6 –** For between-subject designs, place all subject-specific data structures into one set. Randomly select as many data structures as were originally in condition 1 and place these in subset 1. Repeat this process for as many structures that were in condition 2, placing these in subset 2. This process is carried out for all conditions; hence the subject-specific data structures are randomly partitioned, pairing with different conditions. For within-subject designs, random partition is performed per subject, with each subject-specific data structure assigned to a condition.

**Step 7** – For this random partition, calculate the maximum cluster-level statistic, following the process outlined in Steps 1-5.

**Step 8** – Repeat steps 6 and 7 many times, producing a histogram of the maximum cluster-level statistics for each partition. For a two-tailed test, retain both the largest positive and largest negative-going cluster-level statistics, with two histograms constructed. Notably this is a deviation from Maris and Oostenveld (2007), which states the maximum absolute value should be used. However, this approach assumes a symmetric distribution about zero of permuted test statistics, for each random partition, under the null hypothesis, which typically is not the case. Therefore, in practice, it is appropriate to compare the observed cluster-level statistic, with the corresponding positive or negative distribution, providing an appropriate correction is made for the two comparisons performed, e.g., Meyer et al. (2021).

**Step 9** – Using a Monte Carlo estimate, approximate the permutation *p*-value; specifically, this is the proportion of random partitions, that result in a maximum cluster level statistic, larger than the observed statistic. Notably, as stated in step 8, this is performed separately for positive and negative going clusters, with observed statistics evaluated against the corresponding positive or negative distribution. Monte Carlo estimation is required as to calculate the true permutation *p*-value, one would have to repeat steps 6 and 7 an infinite number of times, to construct the permutation distribution.

**Step 10** – If the *p*-value is below the predefined critical α, with applied two-fold multiple comparison correction for two-tailed tests, one can reject the null hypothesis. Notably, the alpha value against which the null hypothesis is evaluated, need not be the same as the threshold for initial cluster entry.

**Step 11** - For effect localisation, select all additional clusters formed in step 3 and evaluate these under the distribution(s) produced in step 8. Critically, irrespective of the size order of the cluster, it must be evaluated against the histogram of maximum cluster-level statistics from the random partitions, to provide control of the false alarm rate for all clusters. For two-tailed tests, this should be either the histogram of maximum positive or negative cluster statistics, depending on the cluster direction. Finally, repeat the procedure in steps 9 and 10 using a Monte Carlo estimate to approximate a permutation *p*-value for each cluster.

## Parameter Selection

## Chosen test statistic

It is necessary to select an appropriate statistical test for sample-wise comparison; here, the example data is from a within-subject designs, hence a paired samples *t*-test is used.

## Initial cluster entry threshold

A decision must also be made regarding the threshold for initial cluster entry in step 2, i.e., the critical α for sample-wise comparison. Selecting a higher threshold, e.g., α ≤ 0.05, will result in weaker effects that spread across multiple samples being detected, but this is at the expense of spatial sensitivity, with additional samples adjacent to a strong effect included within a cluster, as highlighted by Mensen and Khatami (2013). As free harmonic clustering increases the number of possible neighbours for a given sample, the chances of large diffuse clusters forming increases. Therefore, a stricter cluster entry threshold is appropriate, e.g., α ≤ 0.01.

**Final cluster evaluation threshold**

The choice of the final critical α value, against which the null hypothesis is evaluated, and the localisation procedure performed (step 11), depends on the confidence required in the experimental findings. Here, we have used α ≤ 0.05, as is typically (and arbitrarily) applied in psychology.

**Number of permutations**

As producing a full permutation distribution would require an infinite number of samples, one must decide the number of random partitions performed (step 8). The Monte Carlo *p*-value is binomially distributed, hence a confidence interval for a binomial proportion can be constructed around it (Ernst, 2004). Therefore, sufficient permutations should be performed so that this confidence interval becomes arbitrarily small. While 1,000 permutations would typically be acceptable, in our subsequent examples, 10,000 were performed.
